# The Role of Athlete Support Personnel in Anti-Doping: A Narrative Review of Contemporary Evidence

**DOI:** 10.3390/healthcare14091147

**Published:** 2026-04-24

**Authors:** Iván Martín-Miguel, Millán Aguilar-Navarro, Juan Del Coso, Arturo Franco-Andrés, Carolina García, Alejandro Muñoz

**Affiliations:** 1 Institute of Health and Sport Sciences, Faculty of Health Sciences, Universidad Francisco de Vitoria, 28223 Madrid, Spain; ivan.martinmiguel@ufv.es (I.M.-M.); juan.delcoso@urjc.es (J.D.C.); arturo.franco@ufv.es (A.F.-A.); alejandro.munoz@ufv.es (A.M.); 2 Sport Sciences Research Centre, Rey Juan Carlos University, 28943 Fuenlabrada, Spain; 3 Department of Education, Spanish Commission for the Fight Against Doping in Sport (CELAD), 28016 Madrid, Spain; carolina.garcia@celad.gob.es

**Keywords:** clean sport, doping prevention, ethical decision-making, support network, anti-doping education

## Abstract

Doping remains a major threat to athlete health and sport integrity. Although anti-doping efforts have traditionally focused on athletes, increasing attention has turned to Athlete Support Personnel (ASP) due to their influence on athletes’ decisions, behaviors and involvement in anti-doping rule violations. This narrative review aimed to synthesize the existing literature on the role of ASP (including coaches, physicians, pharmacists, sport psychologists, nutritionists, physiotherapists, parents and other family members) in anti-doping, with particular attention to their influence on athletes’ knowledge, attitudes, behaviors, education and decision-making related to doping. Coaches, physicians, and pharmacists are among the ASP groups most frequently examined in the literature, although substantial knowledge gaps remain across all groups. Coaches shape motivational climates and ethical norms but often lack adequate understanding of anti-doping regulations and supplement risks. Physicians and pharmacists play key roles in medication management and Therapeutic Use Exemptions procedures, though incomplete regulatory knowledge may contribute to inadvertent violations. Nutritionists are central in preventing supplement-related doping, while research on sport psychologists and physiotherapists remains limited despite their preventive potential. Parents significantly shape athletes’ moral development and susceptibility to doping, acting as protective or risk factors depending on family dynamics. Overall, anti-doping education for ASP remains inconsistent. In conclusion, ASP plays an essential yet heterogeneous role in influencing doping-related behaviors. Strengthening role-specific and interdisciplinary anti-doping education, particularly within university programs and professional development, appears critical for enhancing ASP competence and promoting a sustainable culture of clean sport.

## 1. Introduction

Doping is broadly defined within the World Anti-Doping Code as the occurrence of one or more Anti-Doping Rule Violations (ADRVs), which include not only the use of prohibited substances or methods but also behaviors such as possession, trafficking, administration, tampering, and complicity [1]. Despite sustained global anti-doping efforts, doping remains a persistent issue in sport, representing a serious threat to athletes’ health and well-being and a clear violation of the fundamental values underlying the spirit of sport [2].

Estimating the true prevalence of doping behavior remains a challenging task. Official laboratory data reported by the World Anti-Doping Agency (WADA) indicate an analytical finding rate of approximately 1–2% [3]. However, these figures underestimate the actual prevalence of doping, as they only reflect detected cases and fail to capture undetected practices or behaviors that evade current testing methodologies. In contrast, studies using indirect estimation techniques and anonymous survey methods have consistently reported substantially higher prevalence rates. Reviews of available data suggest prevalence estimates ranging from 14% to 39% [4] while studies employing the randomized response technique have reported values as high as 61.8% in specific sports and international competition settings [5]. Considering the large number of athletes participating in organized sports worldwide, even the most conservative estimates suggest that doping affects thousands of athletes globally, with particularly elevated risks reported in certain sports and competitive contexts characterized by strong performance pressures and permissive doping environments [6]. In this context, increasing attention has been directed toward the broader social environment surrounding athletes, particularly the individuals who support and interact with them in their sporting careers.

Among these actors, Athlete Support Personnel (ASP) represent a particularly influential group. According to the World Anti-Doping Code, ASP encompasses a wide range of individuals, including coaches, strength and conditioning specialists, sport directors, representatives, team members, agents, medical and paramedical staff, parents, and any other persons working with or supporting athletes participating in or preparing for sport competitions [3]. Their responsibilities include cooperating with anti-doping organizations, complying with applicable anti-doping regulations, and positively influencing athletes’ values and behaviors to foster a culture of clean sport [3]. This central position within the athlete’s support environment is also reflected in the regulatory framework of the World Anti-Doping Code. Notably, of the eleven ADRVs described in the Code, seven can involve the actions or responsibilities of ASP, underscoring their central role in both safeguarding clean sport and, potentially, facilitating doping practices [3].

The influence of ASP on doping-related behaviors can also be understood through established behavioral and socio-ecological frameworks. Research grounded in social–cognitive approaches indicates that athletes’ attitudes and behaviors regarding doping are shaped not only by individual beliefs but also by observational learning, perceived norms, and interactions with influential figures within their social environment [7,8]. Similarly, the Theory of Planned Behavior highlights the importance of subjective norms and perceived behavioral control in shaping doping intentions, both of which may be influenced by members of the athlete’s support network [9,10]. In sport contexts, motivational climate theory further emphasizes how coaches and other support personnel can shape athletes’ achievement goals, ethical orientations, and performance pressures, which have been associated with attitudes toward doping [11]. Finally, socio-ecological perspectives conceptualize doping as the result of interactions between individual, interpersonal, organizational, and cultural factors, highlighting the importance of the athlete’s entourage in shaping doping-related norms and behaviors [12]. Together, these perspectives support the need to examine ASP not only as providers of technical expertise but also as influential actors in the development of attitudes, norms, and decision-making processes related to doping.

In recent years, anti-doping research and policy have increasingly shifted from viewing doping solely as an individual athlete decision to recognizing it as a phenomenon embedded within broader social and organizational systems. This perspective emphasizes the role of the athlete’s entourage, including coaches, medical staff, family members, and other support personnel in shaping norms, expectations, and decision-making processes related to performance enhancement and doping behaviors [13,14]. Reflecting this systemic approach, the WADA has expanded its prevention strategies beyond testing and sanctions to include education programs and responsibilities targeting ASP through the International Standard for Education [15]. Consequently, understanding the knowledge, attitudes, and practices of ASP has become increasingly important for developing effective and sustainable doping prevention strategies within sport systems.

Despite the recognized importance of ASP in doping prevention, empirical evidence regarding their knowledge, attitudes, and behaviors in the anti-doping context remains limited and fragmented [16]. Accordingly, the aim of the present narrative review was to synthesize the existing literature on the role of ASP (including coaches, physicians, pharmacists, sport psychologists, nutritionists, physiotherapists, parents and other family members) in anti-doping, with particular attention to their influence on athletes’ knowledge, attitudes, behaviors, education and decision-making related to doping. Specifically, this narrative review is based on contemporary evidence on the role of ASP in anti-doping, with emphasis on the most relevant literature available at the time of writing. Following the distinctions outlined by Grant and Booth [17], this article was conceived as a narrative review aimed at providing an integrative overview of the literature on this topic, rather than a systematic review with explicit and reproducible methodological procedures.

## 2. Coaches

Coaches are recognized as key ASP due to their central role in shaping training processes, motivational climates, and ethical standards. Their impact on athletes’ attitudes toward doping is widely recognized in international anti-doping policy and coaching regulations [3]. Coaches often hold a privileged position in athletes’ “worldview” regarding doping [18], and empirical evidence consistently identifies them as key figures influencing doping-related behaviors, particularly when the coach–athlete relationship is close and trustful [13,19,20]. This influence is partly attributable to the significant time coaches and athletes spend together in training and competition [21].

The performance level at which coaches operate may further shape this influence. Coaches in elite or professional contexts face greater performance pressures, higher stakes, and closer scrutiny, which may increase both exposure to doping-related risk situations and perceived responsibility for prevention [22,23]. In amateur and developmental sport contexts, coaches tend to adopt an educational and values-based approach to anti-doping, emphasizing integrity, fair play and ethical decision-making as part of long-term athlete development [24]. These contextual differences underscore the need for level specific anti-doping education and support.

Coaches’ impact may be direct, stemming from their personal characteristics, beliefs, or moral stance toward doping [18,25,26], or indirect, through the motivational climate they create. Empowering climates, defined by autonomy support and task orientation, are associated with lower doping likelihood, mediated by negative attitudes toward doping and higher self-regulatory efficacy. Conversely, disempowering climates characterized by ego involvement and controlling leadership increase susceptibility [27].

Qualitative work also highlights this influence. Kirby et al. [26] describe how a supportive coach acted as a protective factor against doping, with risky behaviors emerging only after the athlete changed training groups. In general, coaches express negative attitudes toward doping, viewing it as cheating and a violation of sporting values [19,28], and perceive it as a widespread, international issue with estimated prevalence around 19% [19,29]. Concerns about insufficient testing frequency reinforce this stance [30].

Despite this generally strong anti-doping orientation, coaches frequently demonstrate limited knowledge of anti-doping regulations, methods, and health consequences [19,31,32]. In elite football, most coaches were unfamiliar with WADA (57.6%), the Prohibited List (84.9%) [33] and health consequences [34], with similarly low awareness of specific control mechanisms such as the Athlete Biological Passport or whereabouts system [29]. These limitations may lead to unintentional non-compliance [20], reduced capacity to address doping issues with athletes [18,20], and a tendency to defer responsibility to medical staff [19].

Restricted involvement in prevention efforts is further explained by the prioritization of performance, low perceived self-efficacy, and the belief that anti-doping is peripheral to coaching duties [19]. As a result, doping-related discussions remain rare in coaching environments [29], despite the fact that coaches may face sanctions when athletes commit violations, implications not always understood or supported by coaches themselves [19,22].

In some cases, insufficient knowledge and professional pressures may contribute to unethical behaviors or “willful blindness” [35], with a minority of coaches admitting to providing prohibited substances (e.g., 4.5% in García-Grimau et al. [31]). Job security closely tied to athlete performance has been suggested as a contributing factor [36]. Nevertheless, such behaviors appear uncommon. Most coaches embed clean sport principles within a broader coaching philosophy that prioritizes effort, development, and commitment [22].

Coaches also express strong support for mandatory prevention programs, stricter sanctions for facilitators of doping [19], and regular, role-specific anti-doping education [30]. Evidence indicates that motivationally based educational initiatives are effective in fostering anti-doping cultures and promoting need-supportive coaching behaviors [37]. The importance of embedding these initiatives within formal legislation and continuous professional development frameworks has been widely emphasized [29].

Compared with coaches, scientific evidence on strength and conditioning coaches (S&C) and personal trainers remains more limited. Data from Zmuda-Pałka et al. [38] indicate predominantly negative attitudes toward doping among S&C students and personal trainers, although doping is perceived as widespread (88.51%). Most participants (87.14%) believe that high performance can be achieved without doping, yet some conceptual inconsistencies persist. Ethical arguments, such as unfairness (25%), violation of fair play (16%), or cheating (11%), were the primary reasons for rejecting doping, while fewer participants cited legal (6%) or health-related (3%) concerns. Notably, 10.13% viewed doping as necessary for reaching very high performance levels [38].

Overall, coaches play an important role in shaping athletes’ ethical orientations and responses to doping through their close daily interaction. Ensuring adequate anti-doping education and ethical engagement among coaches therefore remains an important consideration within the anti-doping system.

## 3. Physicians

Physicians play an important role within the sport environment by overseeing athletes’ medical care and supporting health-related decision-making in line with clinical and ethical standards. Their relevance within the anti-doping system is particularly linked to the management of medical treatments, therapeutic use exemptions, and medication-related decisions throughout the training and competition process [39]. As licensed medical professionals, they are responsible for prescribing medications in accordance with the WADA Prohibited List and ensuring that treatments provided to athletes comply with applicable anti-doping regulations [40]. In this context, several Anti-Doping Rule Violations (ADRVs) described in the World Anti-Doping Code directly involve medical personnel, including the administration or attempted administration of prohibited substances or methods, as well as complicity in doping practices [1]. Consequently, physicians may act both as critical gatekeepers in the prevention of doping and, in cases of misconduct or negligence, as potential facilitators of anti-doping rule violations.

Another key responsibility of sports physicians relates to the management of Therapeutic Use Exemptions (TUEs) [41], which allow athletes to use otherwise prohibited substances when medically justified. Physicians play a central role in diagnosing medical conditions, determining appropriate treatments, and preparing the documentation required for TUE applications [42]. This process requires not only clinical expertise but also familiarity with anti-doping regulations and procedures. Among ASP members, physicians are also considered one of the groups with the highest levels of anti-doping knowledge [12]; however, this knowledge is often incomplete, with notable gaps related to specific regulatory aspects and professional responsibilities in doping prevention and management [14,40,43,44]. Nevertheless, inadequate knowledge of TUE requirements or the Prohibited List may increase the risk of rejected applications or unintended rule violations, highlighting the importance of specific anti-doping education within sports medicine practice [41], as well as in serious health risks and career-threatening consequences for athletes [40].

Beyond their regulatory responsibilities, physicians also play a fundamental role in preventing inadvertent doping. Athletes frequently rely on medical professionals for advice regarding medications, nutritional supplements, and therapeutic treatments [18], making physicians key actors in identifying substances that may contain prohibited ingredients. Through careful medication management, consultation on supplement use, and collaboration with pharmacists and other healthcare professionals [45], physicians can help reduce the risk of unintentional anti-doping rule violations arising from contaminated supplements, improperly prescribed medications, or inadequate awareness of prohibited substances.

At the same time, the position of sports physicians within elite performance environments may present complex ethical dilemmas [16,46,47,48]. These authors indicate that, while the primary professional obligation of physicians is to protect athletes’ health and well-being, the competitive context of high-performance sport can create pressures related to performance optimization and rapid return to play. In some situations, the medical expertise that enables physicians to safeguard athlete health may also provide knowledge that could be misused to enhance performance through pharmacological means [47,49]. Beyond explicit pressures, physicians may also encounter implicit expectations from coaches or management to prioritize competitive availability over long-term welfare [50]. Clinical decision-making can additionally be influenced, directly or indirectly, by organizational demands, competitive calendars, financial incentives, or entrenched team cultures [51]. In certain contexts, the recurrent use of legally permissible but ethically questionable medical practices may normalize “grey-zone” behaviors that blur the line between legitimate care and performance manipulation [52]. These tensions can generate divided loyalties, as physicians must navigate their duty to the athlete while facing performance-driven expectations from the broader organization [50]. Ultimately, the ethical commitment to protect health and “do no harm” may conflict with the social and institutional pressures embedded within elite sport [47]. Such dilemmas highlight the importance of strong ethical standards and professional integrity within sports medicine [49], as well as the need to expand physicians’ education in anti-doping matters to ensure the provision of safe and appropriate healthcare to athletes [46].

In summary, physicians represent an important component of the anti-doping system due to their involvement in medical decision-making and athlete health protection, highlighting the relevance of appropriate anti-doping knowledge within clinical sport practice.

## 4. Pharmacists

Pharmacists contribute specialized expertise in medication use within the sport context, particularly in relation to pharmaceutical safety, drug interactions, and regulatory classification of substances that may influence athletes’ therapeutic decisions. Their relevance to doping prevention has received growing attention at both national and international levels, given their capacity to support informed and compliant medication practices among athletes [53]. As highly accessible healthcare professionals, pharmacists are frequently consulted by athletes; previous studies indicate that 46.8% of pharmacists receive doping- or medication-related enquiries once a year or less, while 17.0% report receiving such enquiries several times per year [54]. This accessibility places pharmacists in a strategic position to help prevent inadvertent anti-doping rule violations. In particular, pharmacists are well positioned to advise athletes on the risks associated with dietary supplements, which have been identified as a frequent source of inadvertent doping due to contamination with prohibited substances. Nevertheless, evidence consistently shows that although pharmacists generally possess sufficient basic knowledge to direct athletes to reliable information sources and provide initial advice, their level of specialized anti-doping knowledge remains limited [55,56,57], even at the academic level, despite acknowledging their professional responsibility in this area [58]. For example, a study conducted in Norway involving 296 pharmacists reported that 30.6% demonstrated a moderate level of anti-doping knowledge, while 50.8% exhibited a low level of knowledge [59]. Similarly, research in Australia found that approximately 80% of pharmacists lacked the comprehensive training required to effectively prevent anti-doping rule violations and adequately protect athletes’ health [53]. In Taiwan, only 15% of surveyed pharmacists reported having received education or guidance related to substance use in sport [56]. Their professional expertise in pharmacology, medication safety, and drug interactions places pharmacists in a unique position to identify substances included on the Prohibited List and to recommend safer therapeutic alternatives when necessary. However, it is possible that insufficient familiarity with anti-doping regulations, the Prohibited List, and therapeutic use exemption procedures may limit pharmacists’ ability to provide accurate guidance to athletes and other support personnel. These knowledge gaps are particularly concerning given that pharmacists, together with physicians and nutritionists, are among the ASP most frequently involved in anti-doping rule violations, often in relation to medication use and dietary supplements [12].

To address these limitations, Japan has implemented specific certification programs through the Japan Anti-Doping Agency (JADA) for “sports pharmacists”, who receive specialized training in anti-doping regulations and are qualified to provide appropriate guidance on medication use and potential health consequences for athletes [60]. This initiative has been proposed as a reference model for broader international implementation, highlighting the fundamental role of pharmacists, alongside physicians, in delivering safe and effective healthcare to athletes. Overall, pharmacists can play a key role in managing athletes’ legitimate medication needs, preventing inadvertent exposure to prohibited substances, and educating athletes and the general public about the health risks associated with performance-enhancing drugs. Although pharmacists may assume multiple roles in anti-doping efforts [61], there remains a clear need to expand education and training programs related to doping and substance use in sport in order to strengthen their knowledge and professional competencies in this field [57,62].

Strengthening the integration of pharmacists into anti-doping education and healthcare frameworks may help reduce medication- and supplement-related doping risks and support safer therapeutic practices in sport.

## 5. Sport Psychologists

In their professional practice, sport psychologists work with athletes to develop psychological skills such as goal setting, emotional regulation, coping strategies, and ethical decision-making, which are theoretically relevant to how athletes respond to performance pressures and potential doping situations. However, scientific research directly examining the role of sport psychologists as ASP within the anti-doping context remains clearly limited [62]. This lack of direct evidence is apparent even in leading sport psychology journals, such as Psychology of Sport & Exercise, where studies addressing doping from an ASP perspective, and specifically from the standpoint of sport psychologists, are scarce or virtually absent [62].

Indirect evidence from the broader anti-doping and sport psychology literature indicates that psychological variables, including attitudes toward doping, self-regulatory efficacy, moral values, perceived pressure, and motivational climate, are associated with doping susceptibility [63]. In addition, moral disengagement has been identified as a psychological mechanism through which athletes may justify doping behaviors, suggesting that interventions targeting ethical reasoning and moral self-regulation could be relevant for prevention efforts [64]. While these findings do not directly assess sport psychologists’ professional practice in anti-doping, they provide a theoretical basis for considering the potential relevance of psychological expertise in this area.

Based on this indirect evidence, some authors position sport psychologists as strategic agents in the design and implementation of anti-doping education and prevention programs [63]. Moreover, they align with previous research highlighting the need for anti-doping organizations to systematically integrate psychological content and evidence-based strategies into educational initiatives, given their positive effects on athletes’ attitudes and behaviors [65]. Therefore, beyond the need to expand research explicitly addressing sport psychologists as members of ASP within the anti-doping context, the current literature underscores the importance of developing and implementing psychologically informed interventions targeting athletes. Such approaches may enhance doping prevention by complementing traditional strategies focused on regulation and testing, contributing to a more comprehensive and effective framework for protecting clean sport [63].

In sum, sport psychologists can significantly influence athletes’ attitudes, self-regulation, and moral reasoning to support clean sport. Although evidence of their direct role in doping prevention remains limited, existing research highlights their strategic value. Strengthening psychologically informed interventions may therefore effectively complement traditional anti-doping approaches.

## 6. Nutritionists and Dietitians

Nutritionists and dietitians are responsible for providing evidence-based dietary planning and guidance on supplement use, thereby directly influencing athletes’ nutritional practices, recovery strategies, and potential exposure to supplement-related doping risks [63,64]. Within the ASP framework, sports nutritionists are consistently identified as an important source of information regarding nutrition and supplementation, reflecting their high level of domain-specific expertise and frequent interaction with athletes on performance-related decisions [65,66].

This role is particularly relevant given the high prevalence of dietary supplement use among athletes, which has been consistently reported to range between 40% and 70% across different sports and competitive levels [67]. A major concern in this context is the contamination of dietary supplements with prohibited substances. Empirical evidence has demonstrated that a non-negligible proportion of commercially available supplements contain undeclared compounds, including anabolic agents and stimulants [68]. Contamination rates of up to 15% have been reported in certain product categories [68], and more recent analyses continue to identify pharmacologically active substances in supplements marketed to athletes [69]. Given the widespread use of these products, supplement contamination has been repeatedly identified as a significant contributor to inadvertent anti-doping rule violations [70,71].

Beyond supplements, dietary exposure to prohibited substances may also occur through contaminated food sources. A well-documented example is the presence of anabolic agents such as clenbuterol in meat, which has led to adverse analytical findings in athletes without intentional doping behavior [45]. Together, these factors illustrate the complexity of nutritional risk management in sport and emphasize the need for specialized, informed professional guidance.

Within this landscape, nutritionists and dietitians are well positioned to contribute to doping prevention strategies. Their potential impact is particularly critical, given that sports supplementation, often combined with medication use, represents one of the main sources of inadvertent or unintentional doping cases [22,72,73]. Evidence suggests that sports dietitians exhibit greater awareness of supplement contamination risks compared with other ASP, supporting their role in risk mitigation and athlete education [65]. Moreover, athletes frequently rely on these professionals for advice on supplement selection, which may reduce dependence on unverified sources and minimize exposure to high-risk products [66].

At the same time, sports nutritionists operate in highly performance-oriented environments, where athletes often seek nutritional strategies to maximize competitive outcomes. This context requires careful ethical and professional judgment to ensure that recommendations remain fully compliant with anti-doping regulations [74]. Appropriate evaluation, selection, and prescription of supplements, combined with targeted education on high-risk products, position nutritionists as key agents in the prevention of anti-doping rule violations. Given that the WADA annually updates the List of Prohibited Substances and Methods to protect athlete health and sport integrity, maintaining up-to-date regulatory knowledge is an essential component of their professional responsibility [73].

Despite its relevance, research on nutritionists’ role as ASP in anti-doping is limited. This gap calls for examining their preventive role, knowledge, and impact on unintentional doping. Integrating them into anti-doping education could be a valuable strategy, particularly to address supplement-related risks.

## 7. Physiotherapists

Physiotherapists provide essential expertise in injury prevention, rehabilitation, and recovery, maintaining frequent direct contact with athletes during training and competition cycles. Based on this close and sustained presence within the sporting environment, the literature has suggested that physiotherapists may have the potential to contribute to doping prevention initiatives [3]. However, direct empirical research explicitly examining their role as ASP in the anti-doping context remains limited.

Indirect evidence indicates that physiotherapists’ regular involvement in injury management, functional recovery, and the application or recommendation of therapeutic or topical products places them in a sensitive position with respect to the risk of inadvertent anti-doping rule violation [75]. The limited available evidence indicates that physiotherapists often exhibit notable gaps in their knowledge of specific anti-doping regulations, including the Prohibited List, Therapeutic Use Exemptions (TUEs), and doping control procedures [76]. These findings suggest that, in some contexts, insufficient regulatory awareness may constrain their preventive capacity and increase the risk of unintentional non-compliance.

Illustrative cases reported in the public domain further highlight these potential risks. For example, the 2024 case involving professional tennis player Jannik Sinner, in which a positive test for clostebol was attributed to contamination from a topical product applied during physiotherapy treatment, has been cited as an example of how therapeutic practices may carry anti-doping implications when product composition and regulatory status are not fully understood [77]. While no sanction was ultimately imposed, such cases underscore the shared responsibility of athletes and ASP and should be interpreted as contextual illustrations rather than systematic evidence.

Overall, despite their close contact with athletes and preventive potential, physiotherapists face notable limitations in anti-doping education and training. This may reduce their effectiveness and underscores the need for ongoing, professional-specific education. Their sustained interaction with athletes during rehabilitation also offers opportunities to reinforce clean sport values and raise awareness of doping risks.

## 8. Parents and Other Family Members

Family members act as primary socialization agents whose values, expectations, and emotional support shape athletes’ decision-making processes throughout their development. Parents can substantially influence athletes’ attitudes and behaviors toward doping, although the direction of this influence depends largely on parenting style and the broader family environment [78]. Generally, parental presence and support are associated with reduced risk behaviors and healthier development during adolescence [79]. In youth sport contexts, parents also play a fundamental role in the early socialization of ethical values, shaping young athletes’ perceptions of fairness, effort, and acceptable performance-enhancement practices. Interestingly, Erickson et al. [80] identified parents as key agents in deterring doping among young and young adult athletes. This protective effect appears stronger when athletes believe that the use of prohibited substances would negatively affect significant others, particularly their parents [81,82], thereby fostering more negative attitudes toward doping-related behaviors [78]. However, parental influence may also contribute to increased doping risk under certain conditions, placing parents among the groups that can indirectly facilitate doping in adolescent athletes, albeit to a lesser extent [83]. Excessive performance-oriented pressure [84] and an overemphasis on winning and outcomes [82] may lead some athletes to consider the use of performance-enhancing drugs (PEDs) [78]. Accordingly, when adolescents perceive approval of PED use from significant others, the likelihood of engaging in such behavior increases substantially [10,85].

Beyond their general influence on moral development and sport participation, parents may also shape athletes’ doping-related beliefs and decision-making in ways that vary across cultural contexts. Cultural norms regarding achievement, authority, and conformity may influence how parental expectations are expressed and internalized, thereby affecting athletes’ interpretations of performance enhancement and rule compliance [14,86]. In some sporting cultures characterized by strong achievement orientation or hierarchical family structures, parental pressures, whether explicit or implicit, may heighten athletes’ susceptibility to favorable attitudes toward doping as a means of meeting performance expectations [7]. Conversely, in contexts where parental involvement emphasizes autonomy, health protection, and long-term athlete development, parents may function more consistently as protective agents, reinforcing negative attitudes toward doping and encouraging engagement with anti-doping education [78,87]. These culturally embedded patterns suggest that parental influence on doping-related outcomes is not uniform, highlighting the importance of considering cultural context when examining the role of family members in doping prevention efforts [88].

Regarding anti-doping knowledge, parents, like other members of ASP, do not consistently demonstrate optimal levels of understanding. Nevertheless, some studies indicate that a considerable proportion of parents achieve adequate general knowledge following targeted assessments or educational exposure [89]. This knowledge varies according to factors such as gender, age, and sporting background [14]. For example, fathers have been shown to possess greater knowledge of doping and its side effects than mothers, and parents with prior sporting experience tend to hold different attitudes toward doping compared with those without such experience [89].

Overall, these findings highlight the key role of the parent–athlete relationship in shaping values and attitudes toward doping within the family environment [89]. Family-based interventions may therefore serve as an effective complement to doping prevention. In particular, educating parents of young athletes could reinforce clean sport values early and reduce the risk of doping-related attitudes later in athletes’ careers [90].

**Figure 1:**
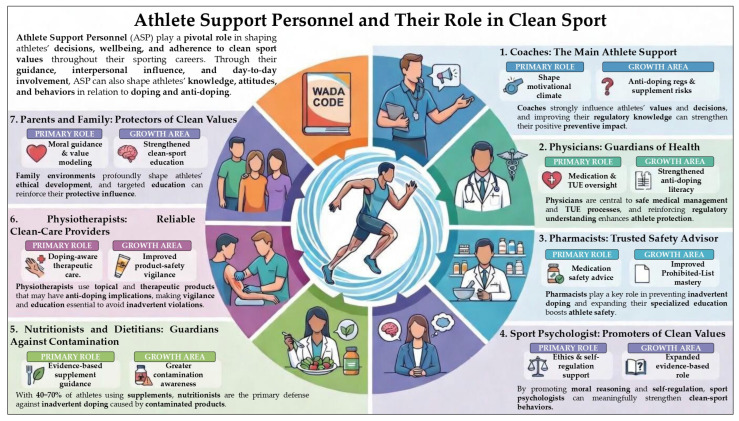
Infographic illustrating the role of athlete support personnel (ASP) in anti-doping, highlighting the main professional groups involved in the athlete’s environment and their potential influence on doping prevention and doping-related risk situations.

An infographic depicting the conceptual role of each ASP within the anti-doping system is presented in Figure 1. This figure serves as an illustrative aid to clarify the multidimensional relationships discussed in the text, rather than as an evidentiary or comparative representation of the strength of evidence across ASP groups.

## 9. Anti-Doping Education for Athlete Support Personnel

Given the knowledge gaps identified across several groups of ASP, education has emerged as a central pillar of contemporary anti-doping strategies. In recent years, WADA has increasingly emphasized prevention and education alongside traditional detection-based approaches. This shift is reflected in the implementation of the International Standard for Education, which establishes guidelines for the development of values-based and knowledge-based education programs targeting athletes and ASP [1]. Within this framework, ASP are recognized as key stakeholders in fostering a culture of clean sport, as their professional responsibilities place them in positions where they can influence athletes’ knowledge, attitudes, and decision-making processes related to doping. Consequently, several studies have highlighted the importance of integrating anti-doping education into university curricula and professional training programs, particularly for future coaches, sports scientists, healthcare professionals, and other practitioners working with athletes [91,92,93].

In addition to formal academic education, continuing professional development initiatives (for instance, coach certification programs, medical training, and specialized professional workshops) have been proposed as effective strategies to strengthen ASP competence in anti-doping matters [20,62]. In practice, anti-doping education is increasingly delivered through online platforms, such as the Anti-Doping Education and Learning (ADEL) platform and educational programs implemented by National Anti-Doping Organizations (NADOs). However, participation in many of these initiatives remains largely voluntary, which may limit their reach, particularly among those ASP who may be most in need of structured anti-doping education.

In this context, it may be valuable to consider whether minimum education requirements or certification schemes could be further explored for ASP working with athletes in high-performance environments. For example, it could be beneficial to examine the potential impact of requiring evidence of basic anti-doping education or certification for ASP accompanying athletes to major competitions, or those working with elite and developing athletes, as a way to promote a more consistent baseline of knowledge regarding the Prohibited List, therapeutic use exemptions, supplement-related risks, and professional responsibilities. Such approaches, while not yet consistently supported across all contexts, may offer a useful direction for future policy development and research. Overall, strengthening anti-doping education among ASP appears to be an important component of comprehensive and sustainable doping prevention strategies in sport.

Table 1 provides a schematic overview of the main ASP categories addressed in this review and their potential roles in doping prevention and risk contexts, based on the narrative synthesis of the literature.

## 10. Limitations and Future Directions

Although this narrative review synthesizes the available literature on the role of ASP in the anti-doping context, several conceptual limitations within the current evidence base should be acknowledged. In addition, as this is a narrative rather than a systematic review, it allows for maintaining a broader contextual understanding of the topic but may be more susceptible to selection bias, lack of reproducibility, and less exhaustive coverage of the available evidence. Research on coaches remains the most extensive, yet it has predominantly examined their knowledge of anti-doping regulations, while broader aspects such as value transmission, competitive pressure, and their influence on athletes’ decision-making processes are still insufficiently explored. Similarly, studies involving medical personnel have largely focused on general anti-doping knowledge, with limited attention to practice-related issues such as TUE management or decisions involving potentially prohibited medications. Other ASP groups, including nutritionists, sport psychologists, and members of the athlete’s close environment, have mostly been examined through declarative knowledge, leaving behavioral, ethical, and contextual dimensions comparatively overlooked. Several relevant professional groups, such as physiotherapists, rehabilitation staff, agents, and managers, also remain underrepresented in the literature. Future research should move beyond predominantly knowledge-based assessments to examine how ASP influence athletes’ ethical decision-making, social norms, and susceptibility to doping in real sporting environments. Expanding the scope to incorporate multidimensional perspectives that capture behavioral, ethical, and contextual factors would help address existing gaps in the literature. Greater attention to social, organizational, and contextual influences may support the development of more comprehensive and context-sensitive prevention strategies that better reflect the complex systems surrounding athletes.

## 11. Conclusions

Overall, this review highlights the central role of ASP within the anti-doping system, as their professional responsibilities place them in a position to influence athletes’ knowledge, attitudes, and decisions regarding doping. Although most ASP express strong opposition to doping and support the principles of clean sport, the literature consistently identifies important gaps in anti-doping knowledge, training, and perceived professional responsibility across several professional groups. These limitations may increase the risk of both intentional and inadvertent anti-doping rule violations, particularly in areas such as medication use, supplement consumption, and ethical decision-making in high-performance environments.

From a practical and policy perspective, strengthening anti-doping education among ASP appears to be an important consideration for effective and sustainable doping prevention. In addition to expanding university curricula and continuing professional development initiatives, the review suggests that exploring approaches such as recommended minimum education standards or voluntary certification schemes for ASP working with elite and developing athletes may be of value. For example, encouraging evidence of basic anti-doping education for ASP accompanying athletes to major competitions could help promote a more consistent baseline of knowledge and reinforce shared responsibility for protecting clean sport. These proposals should be interpreted as implications informed by the narrative synthesis, rather than as evidence-established requirements.

At the same time, future research should move beyond knowledge-based assessments and further examine how ASP influence athletes’ ethical decision-making, social norms, and susceptibility to doping within real sporting environments. Such research would contribute to the development of more comprehensive and context-sensitive prevention strategies that recognize the broader social and organizational systems surrounding athletes.

## Figures and Tables

**Table 1:** Summary of the role of Athlete Support Personnel (ASP) in anti-doping.

ASP Group	Role	Influence on Doping Behaviors	Strength of Evidence	Strengths	Gaps	Implications for Prevention
Coaches [3,13,18,19,20,21,22,23,24,25,26,27,28,29,30,31,32,33,34,35,36,37,38]	Lead training process, shape motivational climate, daily interaction with athletes	Strong influence on values, performance expectations and attitudes toward doping	Strong	Generally negative stance toward doping; recognized role in clean sport promotion	Knowledge gaps in anti-doping regulations and supplements; influence may reinforce performance pressure	Integrate anti-doping education into coach certification and continuous professional development
Physicians [1,12,14,16,18,39,40,41,42,43,44,45,46,47,48,49,50,51,52]	Provide medical care, prescribe medications, manage TUEs	Key role in medication management and therapeutic exemptions	Moderate-strong	Strong awareness of athlete health protection	Risk of inadvertent doping due to medication or supplement advice	Improve anti-doping education in medical training and sports medicine programs
Pharmacists [12,53,54,55,56,57,58,59,60,61,62]	Advice on medications and supplement use	Potential to prevent inadvertent doping through medication guidance	Moderate	Positioned to identify prohibited substances	Limited awareness of the Prohibited List in some contexts	Promote specialized training such as sports pharmacy programs
Sport psychologists [88,94,95,96]	Support mental performance, motivation and coping strategies	Influence on ethical decision-making and motivational climate	Inferential	Potential to reinforce ethical values and self-regulation	Very limited research on their role in anti-doping	Integrate anti-doping ethics and decision-making training in sport psychology education
Nutritionists/dietitians [12,22,45,63,64,65,66,67,68,69,70,71,72,73,74]	Provide dietary and supplementation advice	Influence supplement use and nutrition practices	Moderate	Promote evidence-based nutrition strategies	Risk of supplement contamination and insufficient anti-doping knowledge	Strengthening education on supplement risks and prohibited substances
Physiotherapists [3,75,76,77]	Injury prevention and rehabilitation	May handle topical medications or treatments	Limited	Frequent contact with athletes during injury management	Limited awareness of anti-doping implications of treatments	Improve anti-doping awareness in physiotherapy education
Parents/family [7,10,14,78,79,80,81,82,83,84,85,86,87,88,89,90]	Early socialization and value transmission, especially in youth sport	Influence moral development and attitudes toward fair play	Moderate	Can promote ethical sport values and long-term athlete development	Understudied role in anti-doping research	Include parents in educational initiatives for youth sport environments

Note. The overall level of support reflects the breadth, consistency, and directness of the literature identified in this narrative review by the authors and should be interpreted as a qualitative summary rather than a formal evidence-certainty grading.

## Data Availability

No new data were created or analyzed in this study. Data sharing is not applicable to this article.
